# Supplementary material for: Association of pre-existing comorbidities with mortality and disease severity among 167,500 individuals with COVID-19 in Canada: A population-based cohort study
Source: PLoS One. 2021 Oct 5;16(10):e0258154. doi: 10.1371/journal.pone.0258154 (PMC8491945; doi:10.1371/journal.pone.0258154)
Supplement: S1 Appendix — (DOCX) [file pone.0258154.s001.docx]

S1 Appendix. List of definitions of comorbidities

| **Comorbidity** | **Case definition** | **Codes** | **Validation** |
| --- | --- | --- | --- |
| Asthma | At least one hospitalization admission with a diagnosis of asthma or two physician claims of asthma diagnosis in a two-year period. | ICD-9/OHIPDX: 493  ICD-10: J45, J46 | Sensitivity = 81%  Specificity = 81%^[[1]](#footnote-1)^ |
| COPD | At least 1 physician billings within 2 years or one hospitalization with a COPD diagnosis code prior to index date. | ICD-9/OHIPDX: 491, 492, 496  ICD-10: J41, J42, J43, J44 | Sensitivity = 85%  Specificity = 79%^[[2]](#footnote-2)^ |
| Dementia | Over 40 years old, at least one hospitalization admission or one prescription in the Ontario Drug Benefit program for cholinesterase inhibitors or over three physician claims at least 30 days apart in a one-year period | ICD-9: 46.1, 290.0, 290.1, 290.2, 290.3, 290.4, 294.x, 331.0,  331.1, 331.5, 331.82  ICD-10: F00.x, F01.x, F02.x,  F03.x, G30.x  OHIP: 290, 331 | Sensitivity = 79%  Specificity = 99%^[[3]](#footnote-3)^ |
| HIV | At least three physician claims for HIV diagnosis in a three-year period | OHIPDX: 042, 043, 044 | Sensitivity = 96%  Specificity = 99%^[[4]](#footnote-4)^ |
| Hypertension | At least one hospital admission with a diagnosis of hypertension or two physician claims for hypertension within two years was included. Cases of gestational hypertension were excluded. | ICD-9/OHIPDX: 401x, 402x, 403x, 404x, 405x  ICD-10: I10, I11, I12, I13, I15 | Sensitivity = 73%  Specificity = 95%^[[5]](#footnote-5)^ |
| Diabetes | Age <= 18:  At least four physician claims with a diabetes diagnosis code in a two-year period OR at least one physician claims with a diabetes fee code.  Age 19+:  At least one hospital admission with a diagnosis of diabetes or two physician claims for diabetes or one prescription claim for diabetes medications within 1 year. Cases of gestational diabetes were excluded. | ICD-9/OHIPDX: 250  ICD-10: E10, E11, E13, E14  OHIP Feecode: Q040, K029, K0303, K045, K046  ODB DINS: INSULIN or ORAL ANTI-GLYCEMICS | Age <=18:  Sensitivity: 83%  Specificity: 99%  Age 19+  Sensitivity = 90% Specificity = 98%^[[6]](#footnote-6)^ |
| Chronic kidney diseases | At least one hospital admission with a diagnosis of chronic kidney diseases, or one physician claim, or emergency department visit for a diagnosis of chronic kidney diseases within the last five years.  Or receiving at least one dialysis billing code in each of the three months prior to Jan 15, 2020. | Diagnosis code:  ICD-9: 4030; 4031; 4039; 4040; 4041; 4049; 585; 586; 5888; 5889; 2504  ICD-10: E102, E112, E132, E142, I12, I13, N08, N18, N19  OHIP dx: 403, 585  Dialysis code:  CCP: 5195, 6698  CCI: 1PZ21HQBS, 1PZ21HQBR, 1PZ21HPD4  OHIP: R849, G323, G325, G326, G860, G862, G863, G865, G866, G082, G083, G085, G090, G091, G092, G093, G094, G095, G096, G294, G295, G330, G331, G332, G861, G864, G333, H540, H740 | Sensitivity = 33%  Specificity > 94%^[[7]](#footnote-7)^ |
| Cancer | A history of cancer based on at least one diagnosis in the Ontario Cancer Registry (OCR) prior to January 15, 2020. The OCR contains information on all malignant cancers (except for non-melanoma skin cancers) diagnosed in Ontario. It is a passive registry that is comprised of information from 4 data major sources: cancer-related hospital and surgical records (from CIHI DAD and NACRS data); pathology reports; records from Ontario’s Regional Cancer Centers; and death certificates with a mention of cancer. |  | Not validated. |
| Congestive heart failure | At least one hospital admission with a congestive heart failure diagnosis, one physician claim/emergency department visit with a congestive heart failure diagnosis followed within one year by a second record from either source or one hospital admission. | ICD-9/OHIPDX: 428  ICD-10: I500, I501, I509 | Sensitivity = 85%  Specificity = 97%^[[8]](#footnote-8)^ |
| Cardiac ischemic disease | At least one hospitalization admission with a diagnosis of cardiac ischemic diseases (angina, chronic ischemic heart disease, or myocardial infarction) in the past five years.  Or history of receiving the cardiac procedures of coronary artery bypass grafting or percutaneous coronary interventions in the past 20 years.^[[9]](#footnote-9)^ | Diagnosis code  ICD-10: I20, I25, I21, I22  Procedure code:  CCI: 1IJ76, 1IJ50, 1IJ5, 1IJ57  CCP: 481, 4802, 4803 | Not validated |
| Transient ischemic stroke | At least one hospitalization admission or one emergency department visit with a diagnosis of transient ischemic stroke.^[[10]](#footnote-10)^ | Diagnosis code  ICD-9: 435, 3623  ICD-10: G450, G451, G452, G453, G458, G459, H340 | Not validated. |
| Acute ischemic stroke | At least one hospitalization admission or emergency department visit with a main acute ischemic stroke diagnosis. | Diagnosis code  ICD-9: 434, 436  ICD-10: I63 (excluding I63.6), I64, H34.1 | Not validated. |
| Hemorrhagic stroke | At least one hospitalization admission or emergency department visit with a diagnosis of hemorrhagic stroke. | Diagnosis code:  ICD-9: 430, 431  ICD-10: I60, I61 | Not validated. |
| Rheumatoid arthritis | At least one hospitalization admission with a diagnosis of rheumatoid arthritis, or at least three diagnostic codes of rheumatoid arthritis over two years with at least one provided by a musculoskeletal specialist (rheumatology, orthopedic surgery, or internal medicine). | ICD-9/OHIPDX: 714  ICD-10: M05, M06 | Sensitivity = 78%  Specificity = 100%^[[11]](#footnote-11)^ |
| Inflammatory bowel disease | Age <18:  At least one OHIP procedure code for sigmoidoscopy/colonoscopy and at least two hospitalizations or at least four physician billings in OHIP or emergency department visits in a three year period OR at least three hospitalizations or at least seven physician billings in OHIP or emergency department visits in a three year period (if no OHIP procedure)  Age 18-64:  Two years of OHIP eligibility and at least five hospital, emergency department, and/or physician billing records for Crohn’s disease or ulcerative colitis in a four year period OR at least three hospital, emergency department, and/or physician billing records for Crohn’s disease or ulcerative colitis in a four year period (no 2-year OHIP eligibility)  Age 65+:  Two years of OHIP eligibility and at least five hospital, emergency department, and/or physician billing records for Crohn’s disease or ulcerative colitis in a four year period and at least one ODB claim for IBD medication OR at least three hospital, emergency department, and/or physician billing records for Crohn’s disease or ulcerative colitis in a four year period and at least 1 ODB claim for IBD medication (no 2-year OHIP eligibility) | ICD-9/OHIPDX: 555, 556  ICD-10: K50. K51  OHIP procedure Feecode: Z535, Z555, Z580, E740, E741, E7417, E705 | Children/youth (<18):  Sensitivity = 91%  Specificity = 99%^[[12]](#footnote-12)^  Adults (18-64):  Sensitivity = 77%  Specificity = 96%^[[13]](#footnote-13)^  Older adults (65+):  Sensitivity = 59%  Specificity = 99% |
| Liver disease | Patients who have at least one hospitalization admission or two outpatient visits (physician or emergency department visits) with a cirrhosis diagnosis OR patients who have at least one outpatient visit and one hospitalization admission one procedure for decompensated cirrhosis. | Diagnosis code for Cirrhosis  OHIP dx code: 571  ICD-9: 456.1, 571.2, 571.5  ICD-10: I85.9, I98.2, K70.3, K71.7, K74.6  Diagnostic code for Decompensated Cirrhosis  ICD-9: 456.0, 456.2, 572.2, 572.3, 572.4, 782.4, 789.5  ICD-10: I85.0, I86.4, I98.20, I98.3, K721, K729, K76.6, K76.7, R17, R18  Procedure codes:  CCI: 1. NA.13.BA-FA, 1.NA.13.BA-X7, 1.NA.13.BA-BD, 1.KQ.76GP-NR, 1.OT.53.HA  CCP: 1006, 6691  OHIP: J057, Z591 | Sensitivity = 88 - 99%  Specificity = 89 - 98%^[[14]](#footnote-14)^ |
| Severe mental illness | Individuals admitted to an acute care or psychiatric facility in the 2 years prior to index date for anxiety, deliberate self-harm, mood disorders (bipolar, depression), obsessive compulsive and related disorders, personality disorders, schizophrenia spectrum and other psychotic disorders, substance-related and addictive disorders, and trauma or stressor-related disorders.^[[15]](#footnote-15)^ | Substance-Related and Addictive Disorders  ICD-9: 291.x (all 291 codes), 292.x (all 292 codes), 303.x (all 303 codes), 304.x (all 304 codes), 305.x. Provisional=16 can be split as sub-groups:  291.x, 303.x, 3050 = ALCOHOL; 3040, 3047, 3055 = OPIOIDS; 292.x, 304 [excl. 3040, 3047], 305 [excl.3050, 3055] = OTHERDRUGS  ICD-10: F10.x-F19.x, Z72.0. Provisional=16  can be split as sub-groups:  F10 = ALCOHOL  F11 = OPIOIDS  F12, F13, F14, F15, F16, F18, F19 = OTHERDRUGS  F17, Z720 = OTHER  Schizophrenia Spectrum and Other Psychotic Disorders  ICD-9: 293.81/82, 295.x (all 295 codes), 297.x (all 297 codes), 298.x (all 298 codes). Provisional=2  ICD-10: F20.81, F20.9, F22, F23, F25, F06.0/1/2, F28, F29. Provisional=2  Mood disorders  ICD-9: 293.83, 296.x (all 296 codes), 300.4x, 301.13, 311.x, 625.4. Provisional=3, 4 Can be split as follows: Bipolar [296.0x, 296.4x, 296.5x, 296.6x, 296.7x, 296.8x, 301.13. provisional=3], Depressive [296.2x, 296.3x, 296.9x, 300.4x, 311.x, 625.4x. provisional=4], Other mood [293.83]  ICD-10: F06.3, F31, F32, F33, F34. Provisional = 3, 4  Can be split as follows: Bipolar [F31, F34.0, F06.33, F06.34], Depressive [F32, F33, F34.81, F34.1, F06.31, F06.32]  Anxiety disorders  ICD-9: 293.84, 300, 300.0x, 300.2x, 309.21, 313.23. Provisional=5  ICD-10: F06.4, F40.0x, F40.1x. F40.2x, F41.0x/1x, F41.8x/9x, F93.0, F94.0. Provisional=5  Trauma/stressor-related disorders  ICD-9: 308.3x, 309, 309.0x, 309.24, 309.28, 309.3x, 309.4x, 309.81, 309.89, 309.9x, 313.89. Provisional=7  ICD-10: F43.0, F43.1. F43.2, F43.8/9, F94.1/2. Provisional=7  OCD & related disorders  ICD-9: 300.3x, 300.7x, 312.39, 698.4x. Provisional=6  ICD-10: F06.8, F42.2x, F42.3, F42.4, F42.8, F42.9, F45.2, F63.3. Provisional=6  Personality disorders  ICD-9: 301, 301.0x, 301.2x, 301.4x, 301.5x, 301.6x, 301.7x, 301.81-3, 301.89, 301.9x 310.1. Provisional=18  ICD-10: F07.x (all F07 codes), F21, F60. Provisional=18 | Not validated. |
| Solid organ transplant | Individuals who had history of solid organ transplant.^[[16]](#footnote-16)^ | History of solid organ transplant, CCP/CCI codes:  1PC85 (Kidney) 1HZ85 (Heart) 1GR85, 1GT85 (Lung) 1HY85 (Heart & lung) 1OA85 (Liver) 1OK85 (Pancreas) 1OB85 (Spleen) | Not validated. |

1. Gershon AS, Wang C, Guan J, Vasilevska-Ristovska J, Cicutto L, To T. Identifying patients with physician-diagnosed asthma in health administrative databases. Canadian respiratory journal 2009; 16(6): 183-8. [↑](#footnote-ref-1)
2. Gershon A, Wang C, Guan J, Vasilevska-Ristovska J, Cicutto L, To T. Identifying individuals with physcian diagnosed COPD in health administrative databases. *COPD: Journal of Chronic Obstructive Pulmonary Disease* 2009; **6**(5): 388-94 [↑](#footnote-ref-2)
3. Jaakkimainen RL, Bronskill SE, Tierney MC, et al. Identification of physician-diagnosed Alzheimer’s disease and related dementias in population-based administrative data: a validation study using family physicians’ electronic medical records. Journal of Alzheimer's Disease 2016; 54(1): 337-49. [↑](#footnote-ref-3)
4. Antoniou T, Zagorski B, Loutfy MR, Strike C, Glazier RH. Validation of case-finding algorithms derived from administrative data for identifying adults living with human immunodeficiency virus infection. PloS one 2011; 6(6): e21748. [↑](#footnote-ref-4)
5. Tu K, Campbell NR, Chen Z-L, Cauch-Dudek KJ, McAlister FA. Accuracy of administrative databases in identifying patients with hypertension. *Open medicine* 2007; **1**(1): e18 [↑](#footnote-ref-5)
6. Lipscombe LL, Hwee J, Webster L, Shah BR, Booth GL, Tu K. Identifying diabetes cases from administrative data: a population-based validation study. *BMC health services research* 2018; 18(1): 1-8. [↑](#footnote-ref-6)
7. Fleet JL, Dixon SN, Shariff SZ, et al. Detecting chronic kidney disease in population-based administrative databases using an algorithm of hospital encounter and physician claim codes. *BMC nephrology* 2013; 14(1): 1-8. [↑](#footnote-ref-7)
8. Schultz SE, Rothwell DM, Chen Z, Tu K. Identifying cases of congestive heart failure from administrative data: a validation study using primary care patient records. *Chronic diseases and injuries in Canada* 2013; 33(3). [↑](#footnote-ref-8)
9. Tu JV, Chu A, Donovan LR, et al. The Cardiovascular Health in Ambulatory Care Research Team (CANHEART) using big data to measure and improve cardiovascular health and healthcare services. *Circulation: Cardiovascular Quality and Outcomes* 2015; 8(2): 204-12. [↑](#footnote-ref-9)
10. Chung H FK, Ferreira-Legere LE, Chen B, Ishiguro L, Kalappa G, Gozdyra P, Campbell T, Paterson JM, Bronskill SE, Kwong JC, Guttmann A, Azimaee M, Vermeulen MJ, Schull MJ. COVID-19 Laboratory Testing in Ontario: Patterns of Testing and Characteristics of Individuals Tested, as of April 30, 2020. Toronto, ON: ICES, 2020. [↑](#footnote-ref-10)
11. Widdifield J, Bernatsky S, Paterson JM, et al. Accuracy of Canadian health administrative databases in identifying patients with rheumatoid arthritis: a validation study using the medical records of rheumatologists. Arthritis care & research 2013; 65(10): 1582-91. [↑](#footnote-ref-11)
12. Benchimol EI, Guttmann A, Griffiths AM, et al. Increasing incidence of paediatric inflammatory bowel disease in Ontario, Canada: evidence from health administrative data. Gut 2009; 58(11): 1490-7. [↑](#footnote-ref-12)
13. Benchimol EI, Guttmann A, Mack DR, et al. Validation of international algorithms to identify adults with inflammatory bowel disease in health administrative data from Ontario, Canada. Journal of clinical epidemiology 2014; 67(8): 887-96. [↑](#footnote-ref-13)
14. Lapointe-Shaw L, Georgie F, Carlone D, et al. Identifying cirrhosis, decompensated cirrhosis and hepatocellular carcinoma in health administrative data: a validation study. PLoS One 2018; 13(8): e0201120 [↑](#footnote-ref-14)
15. Chung H FK, Ferreira-Legere LE, Chen B, Ishiguro L, Kalappa G, Gozdyra P, Campbell T, Paterson JM, Bronskill SE, Kwong JC, Guttmann A, Azimaee M, Vermeulen MJ, Schull MJ. COVID-19 Laboratory Testing in Ontario: Patterns of Testing and Characteristics of Individuals Tested, as of April 30, 2020. Toronto, ON: ICES, 2020 [↑](#footnote-ref-15)
16. Chung H FK, Ferreira-Legere LE, Chen B, Ishiguro L, Kalappa G, Gozdyra P, Campbell T, Paterson JM, Bronskill SE, Kwong JC, Guttmann A, Azimaee M, Vermeulen MJ, Schull MJ. COVID-19 Laboratory Testing in Ontario: Patterns of Testing and Characteristics of Individuals Tested, as of April 30, 2020. Toronto, ON: ICES, 2020. [↑](#footnote-ref-16)
